# Coexisting Attractors and Multistability in a Simple Memristive Wien-Bridge Chaotic Circuit

**DOI:** 10.3390/e21070678

**Published:** 2019-07-11

**Authors:** Yixuan Song, Fang Yuan, Yuxia Li

**Affiliations:** Key Laboratory for Robot & Intelligent Technology of Shandong Province, Shandong University of Science and Technology, Qingdao 266590, China

**Keywords:** chaos, memristor, Wien-bridge, coexisting attractors, DSP

## Abstract

In this paper, a new voltage-controlled memristor is presented. The mathematical expression of this memristor has an absolute value term, so it is called an absolute voltage-controlled memristor. The proposed memristor is locally active, which is proved by its DC *V*–*I* (Voltage–Current) plot. A simple three-order Wien-bridge chaotic circuit without inductor is constructed on the basis of the presented memristor. The dynamical behaviors of the simple chaotic system are analyzed in this paper. The main properties of this system are coexisting attractors and multistability. Furthermore, an analog circuit of this chaotic system is realized by the Multisim software. The multistability of the proposed system can enlarge the key space in encryption, which makes the encryption effect better. Therefore, the proposed chaotic system can be used as a pseudo-random sequence generator to provide key sequences for digital encryption systems. Thus, the chaotic system is discretized and implemented by Digital Signal Processing (DSP) technology. The National Institute of Standards and Technology (NIST) test and Approximate Entropy analysis of the proposed chaotic system are conducted in this paper.

## 1. Introduction

A memristor is a nonlinear two-terminal circuit element reflecting the relationship between charge and magnetic flux, which was first predicted by Chua in 1971 [1]. Reference [2] introduced the general definition of memristor and its three fingerprints. Then, in 2008, the realization of a nanoscale memristor was first reported by the Hewlett-Packard laboratory [3]. A locally active memristor was proposed by Chua, which can generate complex behaviors in nonlinear dynamical systems [4]. The DC *V*–*I* plot is a smooth curve passing through different voltages *V* and corresponding currents *I* on the *V*–*I* plane, which can be used to show that a memristor is locally active [5]. Because of its unique properties, a memristor can be used in many areas such as nonlinear chaotic circuits [6,7,8,9], artificial intelligence [10,11,12], electronic engineering [13,14,15], neural networks [16,17,18], and so on.

Recently, much attention has been paid to construct memristor-based chaotic circuits and analyze their dynamical behaviors. Reference [19] presented and analyzed a new chaotic circuit, which was composed of a meminductor emulator and an active memristor emulator. Reference [20] constructed a memristor-based hyperchaotic Wien-bridge oscillator and analyzed its dynamical behaviors. In Reference [21], an inductor-free chaotic circuit containing two memristors was proposed. A Wien-bridge chaotic oscillator based on an SBT memristor was designed in Reference [22]. Besides, some dynamical behaviors in chaotic systems were analyzed with the help of a phase diagram, Poincare section, bifurcation diagram, and Lyapunov exponent spectrum [23,24,25]. Specifically, coexisting attractors and multistability are common phenomena in a chaotic system, which indicates that a chaotic system with fixed parameters under different initial conditions can generate disparate attractors. In recent years, many studies have been conducted on coexisting attractors and multistability in chaotic systems. Reference [26] discovered coexisting singular attractors in a two-dimensional dynamical system. The two-dimensional dynamical system consisted of a bistable bi-local active memristor and an inductor. Reference [27] introduced a new four-wing chaotic system and analyzed its multistability. In Reference [28], the multistability phenomenon was detected and analyzed in an autonomous hyperchaotic oscillator. A sustained chaos state means that independent of the initial conditions, a system will finally turn to a chaotic state with constant Lyapunov exponents. The sustained chaos phenomenon was discovered in a new memristor-based chaotic system [29]. Reference [30] proposed a novel 4D chaotic system with constant Lyapunov exponents. In Reference [31], the constant Lyapunov exponent spectrum was found in a Wien-bridge chaotic oscillator based on a meminductor. 

Furthermore, the chaotic system can be used for encryption and secure communication. In Reference [32], a new 3D system was used for signal encryption. A fractional-order hperchaotic system was applied to a color image encryption in Reference [33]. Reference [34] applied a four-dimensional hyperchaotic system to image encryption. Reference [35] designed a chaotic system with adaptive control synchronization and applied it to secure communication. Reference [36] introduced a new 3D autonomous chaotic oscillator and described its secure communication application. The chaotic system can be implemented by a digital circuit, which makes it better applicable to digital encryption. Therefore, the chaotic system can be further discretized and implemented by Digital Signal Processing (DSP) technology [37]. Besides, the chaotic system can be used as a pseudo-random sequence generator to provide key sequences for encryption systems. The randomness of the chaotic sequence can be tested by means of the National Institute of Standards and Technology (NIST) test suite [38].

In this paper, an absolute voltage-controlled memristor is presented. Then, a simple three-order Wien-bridge circuit is constructed based on the presented memristor. Because of the absence of an inductor, this Wien-bridge circuit is easily integrated. In addition, this chaotic system possesses dynamical behaviors, including multistability and sustained chaos state. The rest of this paper is composed of the following sections. In Section 2, the model of this absolute voltage-controlled memristor is presented and its characteristics are researched via a DC *V*–*I* plot. A simple three-order memristive Wien-bridge circuit and its typical chaotic attractors are demonstrated in Section 3. The rich dynamical behaviors of the presented system are analyzed in Section 4. In Section 5, an analog circuit of this chaotic system is realized by the Multisim software. The DSP implementation of this chaotic system is introduced in Section 6. NIST test and Approximate Entropy analysis of the proposed chaotic system are conducted in Section 7. Finally, some conclusions are drawn in Section 8.

## 2. Mathematical Model and DC *V*–*I* Plot of the Proposed Memristor 

### 2.1. Mathematical Model 

According to the definition of memristor, an *n*th-order voltage-controlled memristor can be described by the following equation [1,39,40]:

(1) $$\left\{ \begin{array}{l} i=W(z)v\\ \frac{dz}{dt}=f(z,v) \end{array}\right.$$

where *i* represents the current flowing through the memristor, *v* stands for the voltage across the memristor, *z* is the state variable of the memristor, and *W*(*z*) is the corresponding memductance. 

In order to research the characteristics of a new memristor model and explore the dynamic behaviors of an oscillator system based on the memristor, a new voltage-controlled memristor was proposed as follows:

(2) $$\left\{ \begin{array}{l} i=(a_{m}-b_{n}\left|z\right|)v\\ \frac{dz}{dt}=-cz-dv^{2} \end{array}\right.$$

where *a*_m_, *b*_n_, *c*, and *d* are coefficients, and *a*_m_-*b*_n_|*z*| is the memductance *W*(*z*). The mathematical expression of this memristor has an absolute value term, so it is called an absolute voltage-controlled memristor.

**Figure 1:**
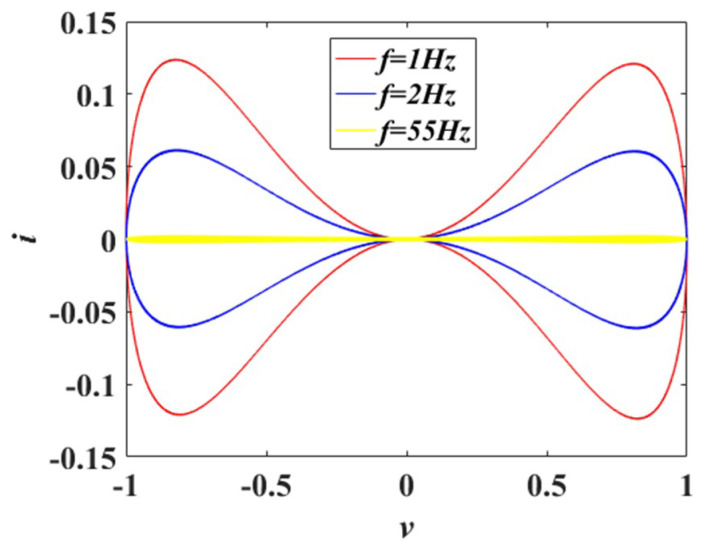
The *v*–*i* pinched hysteresis loops of the proposed memristor with different frequencies *f*.

When a voltage signal *v* = *v*_m_sin(2π*f*t) with amplitude *v*_m_ and frequency *f* is applied to the memristor, the *v*–*i* pinched hysteresis loops of the proposed memristor with amplitude *v*_m_ = 1 V and different frequencies *f* are depicted in Figure 1.

In Figure 1, it is obvious that the *v*–*i* pinched hysteresis loops of the proposed memristor pass through the origin. Besides, the *v*–*i* pinched hysteresis loops are symmetrical. The area of the *v*–*i* hysteresis loop decreases when the frequency *f* increases. The pinched hysteresis loop shrinks to a straight line when the frequency *f* is 55 Hz. Therefore, the proposed memristor satisfies the characteristic fingerprints of memristors [2].

### 2.2. DC V–I Plot of the Proposed Memristor 

The DC *V*–*I* plot is a smooth curve passing through different voltages *V* and corresponding currents *I* on the *V*–*I* plane, which can be used to research the characteristics of a memristor [5]. When the state variable *z* is *Z*, the DC voltage is *V*, and the DC current is *I*, the Equation (2) can be rewritten as follows:

(3a) $$I=(a_{m}-b_{n}\left|Z\right|)V$$

(3b) $$\frac{dZ}{dt}=-cZ-dV^{2}$$

when the right-hand side of (3b) is zero, the equilibrium equation of the memristor can be written as follows:

(4) $$-cZ-dV^{2}=0$$

The relationship between the state variable *z* and the DC voltage *V* can also be written as follows:

(5) $$Z=-\frac{d}{c}V^{2}$$

When *a*_m_ = 5, *b*_n_ = 4, *c* = 0.4, *d* = 1, and Equation (5) is taken into Equation (3a), the DC current *I* can be written as follows:

(6) $$I=(a_{m}-b_{n}\left|\frac{d}{c}V^{2}\right|)V=(5-10V^{2})V$$

**Figure 2:**
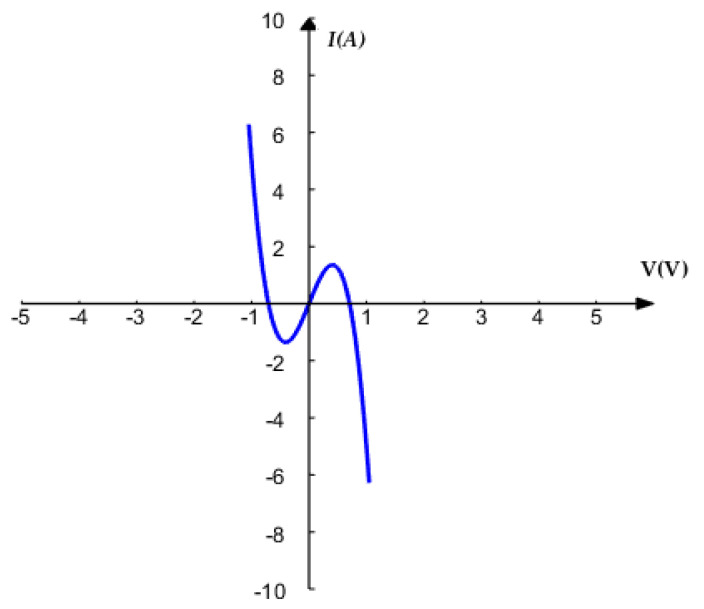
DC *V*–*I* plot of the proposed memristor.

According to Equation (6), the DC *V*–*I* plot of the proposed memristor is shown in Figure 2.

It is obvious that there are negative slope regions in the DC *V*–*I* plot of the memristor. Hence, the proposed absolute memristor is locally active [41]. In a nonlinear dynamical system, the function of a locally active element is to maintain oscillations [41]. 

## 3. The Three-Order Memristive Wien-Bridge Chaotic Circuit

### 3.1. Circuit Model 

**Figure 3:**
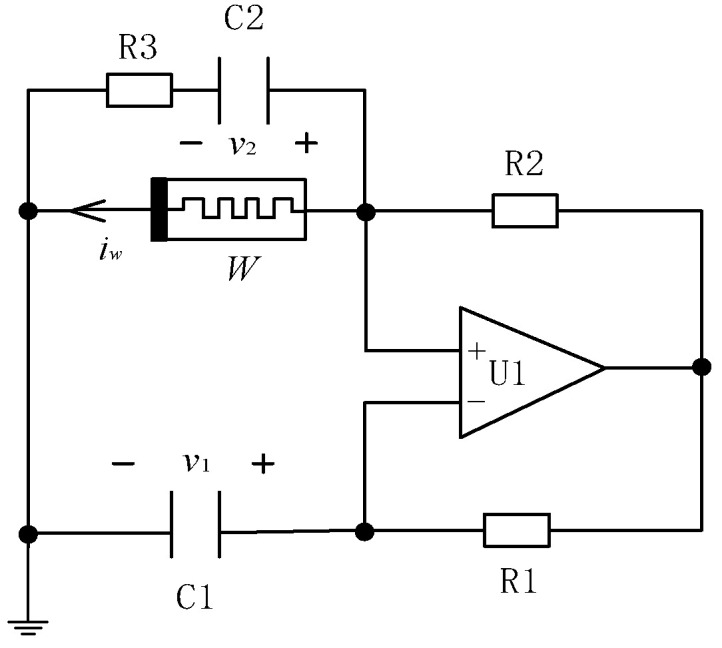
The simple memristive Wien-bridge circuit.

As shown in Figure 3, a simple memristive Wien-bridge circuit based on the above absolute voltage-controlled memristor was constructed. The three-order chaotic circuit consisted of three dynamic elements, i.e., the capacitor C1, the capacitor C2, and the absolute voltage-controlled memristor *W* corresponding to the three state variables voltage *v*_1_, voltage *v*_2,_ and current *i*_w_, respectively. 

According to Kirchhoff’s law and the constitutive relations of circuit elements, the state equations of the above Wien-bridge circuit are written as follows:

(7) $$\left\{ \begin{array}{l} \frac{dv_{1}}{dt}=\frac{R_{2}}{C_{1}R_{1}}[\frac{v_{1}-v_{2}}{R_{3}}+W(z)v_{1}]\\ \frac{dv_{2}}{dt}=\frac{v_{1}-v_{2}}{C_{2}R_{3}}\\ \frac{dz}{dt}=-cz-dv_{1}^{2} \end{array}\right.$$

where *W*(*z*) = *a*_m_ − *b*_n_|*z*|. Let *x* = *v*_1_, *y* = *v*_2_, *R*_1_ = *R*_2_, *a* = *R*_2_/*C*_1_*R*_1_*R*_3_, *b* = *R*_2_/*C*_2_*R*_1_*R*_3_, *m* = *a*_m_/*C*_1_, and *n* = *b*_n_/*C*_1_, Equation (7) can be simplified to:

(8) $$\left\{ \begin{array}{l} \frac{dx}{dt}=a(x-y)+(m-n\left|z\right|)x\\ \frac{dy}{dt}=b(x-y)\\ \frac{dz}{dt}=-cz-dx^{2} \end{array}\right.$$

### 3.2. Typical Chaotic Attractors

**Figure 4:**
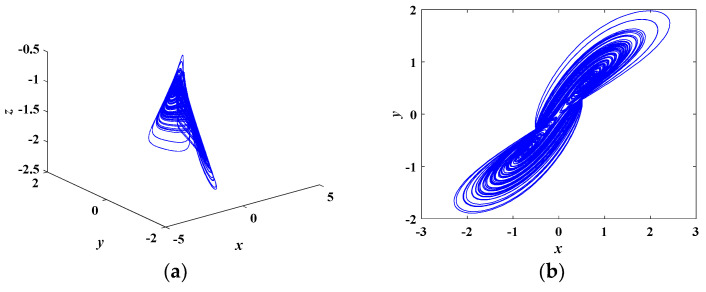

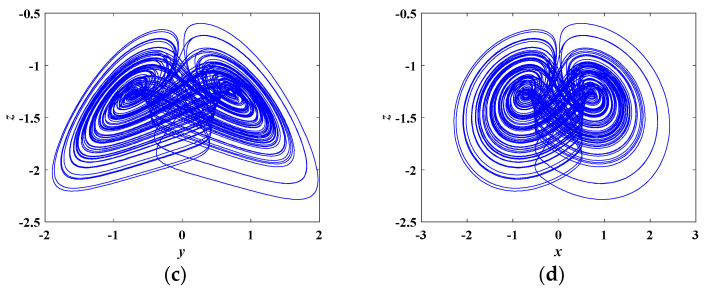
Phase portraits of the proposed chaotic system on (**a**) *x-y-z* plane, (**b**) *x-y* plane, (**c**) *y-z* plane, and (**d**) *x-z* plane.

**Figure 5:**
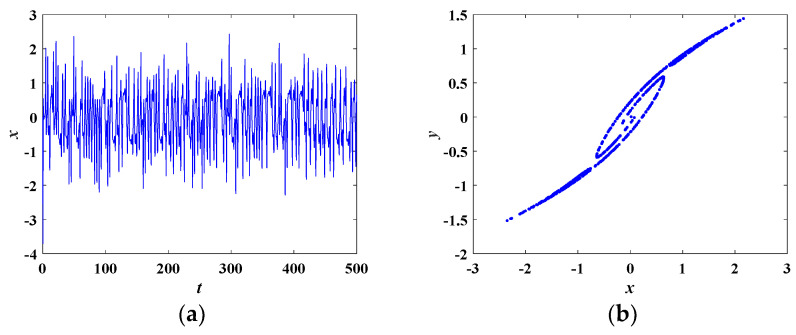
(**a**) The time domain waveform of the state variable *x*, (**b**) the corresponding Poincare mapping on *z* = −1.3 section.

When the parameters of Equation (8) are set as in Table 1 and the initial conditions are (0, 0.1, 0), the system is in a chaotic state. In this condition, the Lyapunov exponents are calculated as *LE*_1_ = 0.4369, *LE*_2_ = 0, *LE*_3_ = −2.0762. The corresponding chaotic attractors on the *x-y-z*, *x-y*, *y-z*, *x-z* planes are depicted in Figure 4. The time domain waveform of the state variable *x*(*t*) is shown in Figure 5a. Figure 5b demonstrates the corresponding Poincare mapping on *z* = −1.3 section. The time domain waveform and Poincare mapping shown in Figure 5 indicate that the system was chaotic.

## 4. Dynamical Behaviors of the Proposed Chaotic System

### 4.1. Dissipativity and Stability

The solutions of Equation (8) are invariant under the following transformation:

(9) (x,y,z)→(−x,−y,−z)

this implies that the proposed chaotic system was symmetric at the origin.

If the system is dissipative, it can generate chaotic attractors. The dissipativity of this system can be described by the following expression:

(10) $$\nabla V=\frac{\partial \dot{x}}{\partial x}+\frac{\partial \dot{y}}{\partial y}+\frac{\partial \dot{z}}{\partial z}=a+m-n\left|z\right|-b-c$$

when the parameters *a*, *b*, *c*, *d*, *m*, and *n* are set as in Table 1 and |*z*| > 0.72, the ∇*V* of this system is negative. It means the system is dissipative.

Let $\dot{x}=\dot{y}=\dot{z}=0$ in Equation (8): three equilibrium points of this system can be calculated as follows:

(11) $$\left\{ \begin{array}{l} S_{0}=(0,0,0)\\ S_{1}=(\sqrt{\frac{mc}{nd}},\sqrt{\frac{mc}{nd}},-\frac{m}{n})\\ S_{2}=(-\sqrt{\frac{mc}{nd}},-\sqrt{\frac{mc}{nd}},-\frac{m}{n}) \end{array}\right.$$

The Jacobian matrix ***J*** of Equation (8) is expressed as follows:

(12) $$J=\left[\begin{array}{ccc} a+m-n\left|z\right| & -a & -nxsign(z)\\ b & -b & 0\\ -2dx & 0 & -c \end{array}\right]$$

If *m* = 5.0 and *n* = 4.0, its characteristic equation at equilibrium point *S*_0_ can be simplified as follows:

(13) $$\det(\lambda I-J)=\lambda^{3}+(b-a-4.6)\lambda^{2}-(0.4a+4.6b+2)\lambda -2b=0$$

In terms of the Routh–Hurwitz stability criterion, if all coefficients of Equation (13) satisfy the following equation, the system is stable:

(14) $$\left\{ \begin{array}{l} b-a-4.6>0\\ -0.4a-4.6b-2>0\\ -2b>0\\ (b-a-4.6)(-0.4a-4.6b-2)+2b>0 \end{array}\right.$$

**Figure 6:**
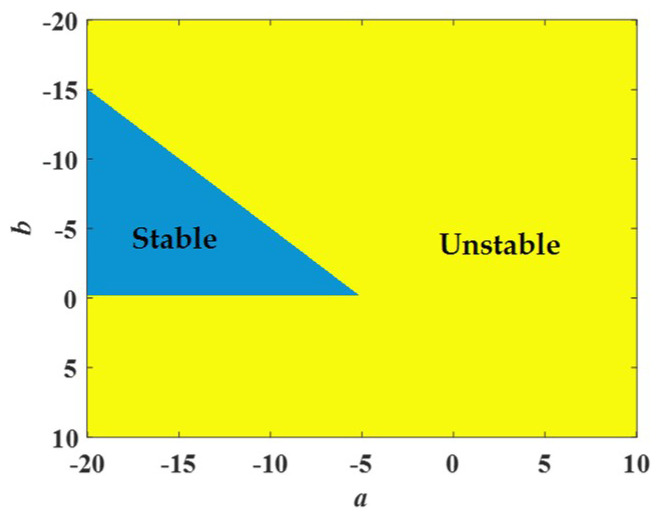
The stable region colored in blue and the unstable region colored in yellow in the region of *a* ∈ [−20, 10] and *b* ∈ [−20, 10].

As shown in Figure 6, the region of *a* ∈ [−20,10] and *b* ∈ [−20,10] can be divided into two parts. The blue part satisfies the above equation, so it represents a stable region. Conversely, the yellow part belongs to an unstable region. 

### 4.2. Bifurcation Diagrams and Lyapunov Exponent Spectra

The dynamical behaviors of the above chaotic system were further investigated with the help of bifurcation diagrams and Lyapunov exponent spectra. 

**Figure 7:**
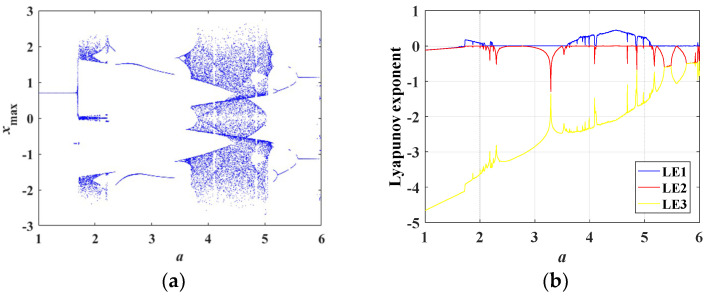
Bifurcation diagram and Lyapunov exponent spectra varying with *a*. (**a**) Bifurcation diagram, (**b**) Lyapunov exponent spectra.

When *a* varied from 1 to 6 and other parameters were set as in Table 1 with initial conditions of (0, 0.1, 0), the bifurcation diagram of the state variable *x* and the corresponding Lyapunov exponent spectra are as plotted in Figure 7a,b, respectively. As it is shown in Figure 7a, when *a* ∈ [1, 1.7], the system was convergent, and the corresponding Lyapunov exponents were all negative. Then, the system went into chaotic state nearby *a* = 1.8, with one of the Lyapunov exponents positive. When *a* ∈ [1.8, 2.2], the system was in chaotic state. Next, the system entered a period window nearby *a* = 2.3. The system was still in periodic state in the regions of *a* ∈ [2.3, 3.4]. In the regions of *a* ∈ [3.5, 5.2], the system was in chaotic state. Finally, nearby *a* = 5.2, the system entered periodic state. 

**Figure 8:**
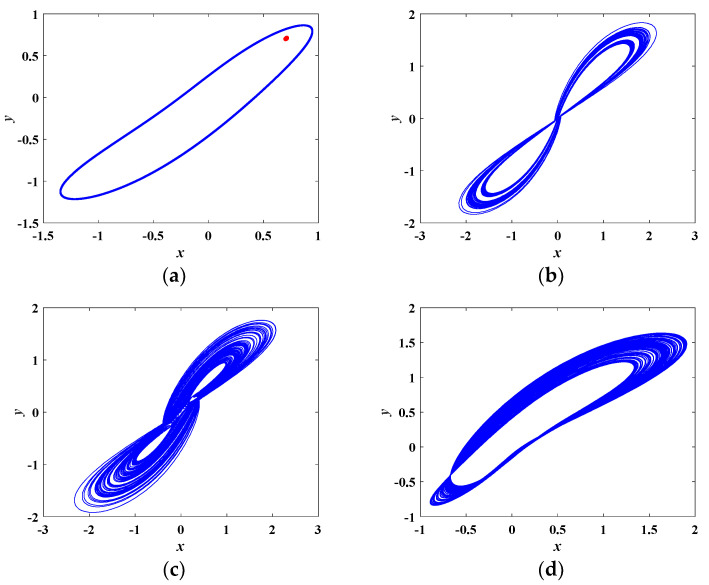
Various phase portraits with different *a* on the *x*-*y* plane. (**a**) *a* = 1.5 in red, *a* = 5.5 in blue, (**b**) *a* = 2.0, (**c**) *a* = 4.0, (**d**) *a* = 5.1.

More specifically, various phase portraits with different *a* are depicted in Figure 8. In Figure 8a, when *a* = 1.5, the red trajectory converges to the stable equilibrium point (0.7071, 0.7071, −1.2500). When *a* = 5.5, the blue trajectory is a limit cycle, implying the system is in periodic state. Different kinds of chaotic attractors are plotted in Figure 8b–d. Figure 8b,c depict two kinds of twin-scroll chaotic attractors. The single-scroll chaotic attractor is shown in Figure 8d. 

### 4.3. Coexisting Attractors and Multistability

Coexisting attractors and multistability are common characteristics of a chaotic system. Generally, if a system possesses coexisting attractors, it has multistability. The existence of coexisting attractors indicates that a system with fixed parameter values and different initial conditions can generate disparate attractors. If a chaotic system has coexisting attractors, it can also show the phenomenon of coexisting bifurcation.

**Figure 9:**
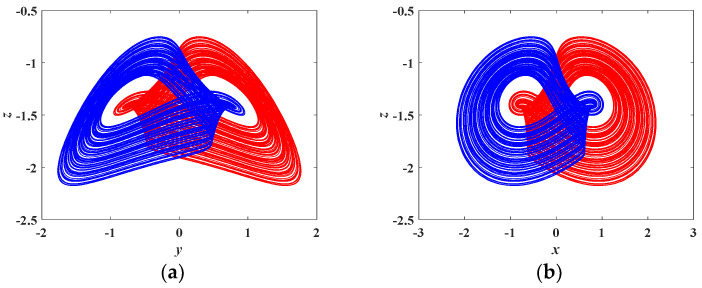
Coexisting attractors, indicated in red at initial conditions of (0, 0.1, 0) and indicated in blue at initial conditions of (0, −0.1, 0). (**a**) coexisting attractors on the *y*-*z* plane (**b**) coexisting attractors on the *x*-*z* plane.

If *a* = 4.5*, b* = 4.85, *c* = 0.4, *d* = 1, *m* = 5, *n* = 4, and the initial conditions are set as (0, 0.1, 0) and (0, −0.1, 0), the coexisting attractors are as depicted in Figure 9, where the red trajectory starts from initial conditions of (0, 0.1, 0), and the blue trajectory starts from initial conditions of (0, −0.1, 0). Obviously, the coexisting attractors starting from (0, 0.1, 0) and (0, −0.1, 0) are symmetric with respect to *y* = 0 on the *y*-*z* plane. The above coexisting attractors are symmetric with respect to *x* = 0 on the *x*-*z* plane. Thus, the coexisting attractors at different initial conditions are symmetrical in this chaotic system. 

**Figure 10:**
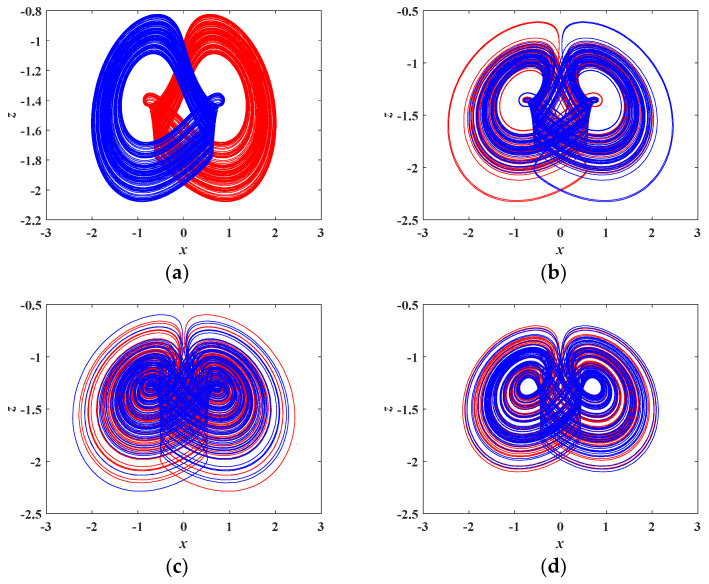

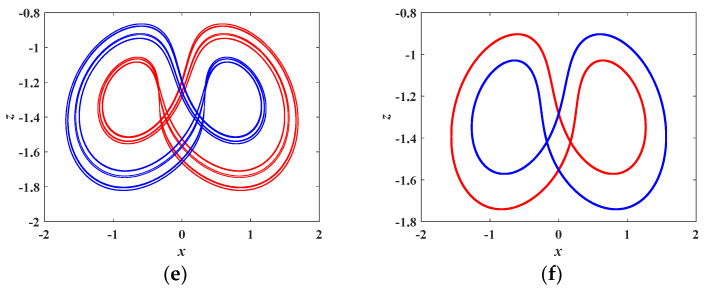
Various coexisting attractors with different values of *b* under initial conditions of (0, 0.1, 0) in red and (0, −0.1, 0) in blue. (**a**) *b* = 4.83, (**b**) *b* = 5.1, (**c**) *b* = 5.5, (**d**) *b* = 5.9, (**e**) *b* = 6.6, (**f**) *b* = 7.0.

When *a* = 4.5, *c* = 0.4, *d* = 1, *m* = 5, *n* = 4, and *b* is set as different values, a variety of coexisting attractors with initial conditions of (0, 0.1, 0) and (0, −0.1, 0) are obtained, as plotted in Figure 10, where the red orbit starts from the initial conditions of (0, 0.1, 0), and the blue one starts from the initial conditions of (0, −0.1, 0). Figure 10a,b depicts two kinds of a symmetric pair of single-scroll attractors. Figure 10c,d depicts two kinds of a symmetric pair of twin-scroll attractors. Two kinds of a symmetric pair of limit cycles are shown in Figure 10e,f.

**Figure 11:**
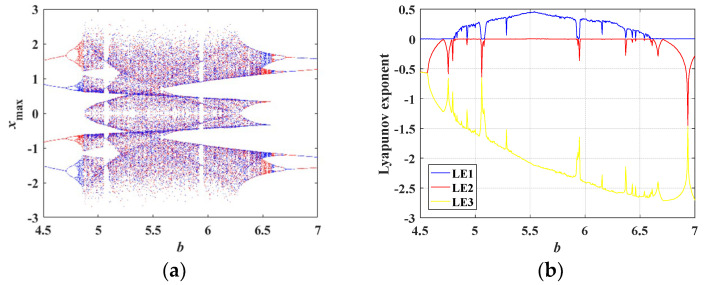
Coexisting bifurcation diagram and the corresponding Lyapunov exponent spectra varying with *b*. (**a**) Coexisting bifurcation diagram of the variable *x* at the initial conditions of (0, 0.1, 0) in red and at the initial conditions of (0, −0.1, 0) in blue, (**b**) corresponding Lyapunov exponent spectra.

When *b* varies from 4.5 to 7 and other parameters are set as in Table 1, the coexisting bifurcation diagram of the state variable *x* is shown in Figure 11a, where the orbit colored in red starts from the initial conditions of (0, 0.1, 0), and the blue one starts from the initial conditions of (0, −0.1, 0). Obviously, the coexisting bifurcation diagram varying with *b* is symmetric with respect to *x*_max_ = 0. The Lyapunov exponent spectra varying with *b* are depicted in Figure 11b and correspond to the coexisting bifurcation diagram plotted in Figure 11a. It can be concluded from Figure 11b that independent of the initial conditions being (0, 0.1, 0) or (0, −0.1, 0), this chaotic system has the same Lyapunov exponents.

### 4.4. Sustained Chaos State

As mentioned above, the proposed chaotic system showed multistability, which means that under different initial conditions, it can always evolve into a chaotic state. Therefore, the proposed chaotic system is very interesting. Independent of the initial conditions, the system will evolve into a chaotic state. This indicates that the proposed system maintains a sustained chaos state and constant Lyapunov exponents as the initial conditions vary. 

**Figure 12:**
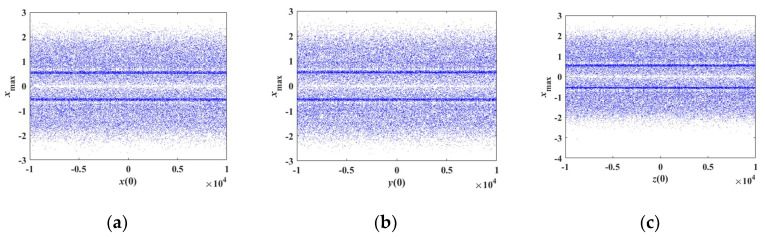
Bifurcation diagram of the state variable *x* varying with different initial values. (**a**) Variation with initial value *x*(0), (**b**) variation with initial value *y*(0), (**c**) variation with initial value *z*(0).

**Figure 13:**
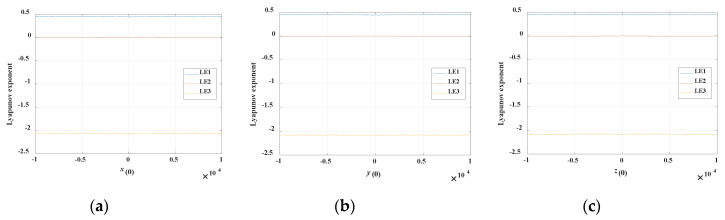
Corresponding Lyapunov exponent spectra varying with different initial values. (**a**) Variation with initial value *x*(0), (**b**) variation with initial value *y*(0), (**c**) variation with initial value *z*(0).

If the system parameters are as in Table 1 with initial conditions of (*x*(0), 0.1, 0) in which *x*(0) is the bifurcation parameter, the bifurcation diagram of the state variable *x* and the corresponding Lyapunov exponent spectra are as shown in Figure 12a and Figure 13a, respectively. It is obvious that the Lyapunov exponents of the proposed system almost remain constant under the initial conditions of (*x*(0) ∈ (−1 × 10^4^, 1 × 10^4^), 0.1, 0). If the initial value *y*(0) is regarded as the bifurcation parameter and *x*(0) = *z*(0) = 0, the bifurcation diagram of the state variable *y* and the corresponding Lyapunov exponent spectra are as shown in Figure 12b and Figure 13b, respectively. The corresponding Lyapunov exponents are approximately the same under the initial conditions of (0, *y*(0) ∈ (−1×10^4^, 1×10^4^), 0). Similarly, the Lyapunov exponents are constant under the initial conditions of (0, 0.1, *z*(0) ∈ (−1×10^4^, 1 × 10^4^)). The bifurcation diagram of the state variable *z* and the corresponding Lyapunov exponent spectra with initial conditions of (0, 0.1, *z*(0) ∈ (−1×10^4^, 1×10^4^)) are shown in Figure 12c and Figure 13c, respectively. 

## 5. Circuit Simulation by the Multisim Software

The circuit simulation of this chaotic system could be realized by the Multisim software [42]. For time scaling factors *τ* = 100*t* and circuit parameters as in Table 1, Equation (8) can be written as follows:

(15) $$\left\{ \begin{array}{l} \frac{dx}{d\tau }=450(x-y)+(500-400\left|z\right|)x\\ \frac{dy}{d\tau }=550(x-y)\\ \frac{dz}{d\tau }=-40z-100x^{2} \end{array}\right.$$

**Figure 14:**
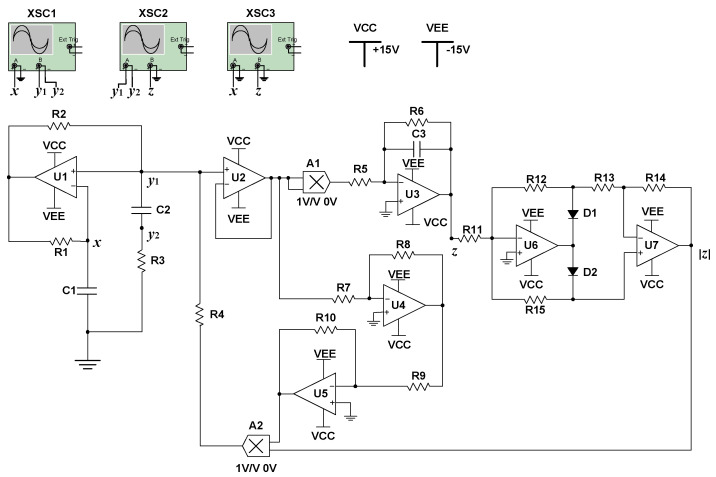
The analog circuit of the memristive Wien-bridge chaotic oscillator.

The analog circuit is shown in Figure 14, from which the state equations can be obtained as follows:

(16) $$\left\{ \begin{array}{l} \frac{dx}{dt}=\frac{1}{C_{1}R_{3}}(x-y)+(\frac{1}{C_{1}R_{4}}-\frac{R_{10}}{C_{1}R_{4}R_{9}}\left|z\right|)x\\ \frac{dy}{dt}=\frac{1}{C_{2}R_{3}}(x-y)\\ \frac{dz}{dt}=-\frac{1}{C_{3}R_{6}}z-\frac{1}{C_{3}R_{5}}x^{2} \end{array}\right.$$

Supposing that the coefficients in Equation (15) are equal to the corresponding ones in Equation (16), the above equations can be written as follows:

(17) $$\left\{ \begin{array}{ll} \frac{1}{C_{1}R_{3}}=450 & \frac{1}{C_{2}R_{3}}=550\\ \frac{1}{C_{3}R_{6}}=40 & \frac{1}{C_{3}R_{5}}=100\\ \frac{1}{C_{1}R_{4}}=500 & \frac{R_{10}}{C_{1}R_{4}R_{9}}=400 \end{array}\right.$$

In Figure 14, the specific capacitance and resistance parameters were set as *C*_1_ = 11 nF, *C*_2_ = 9 nF, *R*_1_ = *R*_2_ = 10 kΩ, *R*_5_ = 4 kΩ, *R*_6_ = 10 kΩ, *R*_7_ = *R*_8_ = 1 kΩ, *R*_9_ = 5 kΩ, *R*_10_ = 4 kΩ, *R*_11_ = *R*_12_ = *R*_13_ = *R*_14_ = *R*_15_ = 10 kΩ. Other capacitance and resistance parameters could be calculated as: *C*_3_ ≈ 2.5 μF, *R*_3_ ≈ 202 kΩ, *R*_4_ ≈ 182 kΩ. In this analog circuit, the type of operational amplifiers U1–U7 was OP07CP, and the type of multipliers A1–A2 was AD633. XSC1, XSC2, and XSC3 were oscilloscopes. D1 and D2 were diodes.

**Figure 15:**
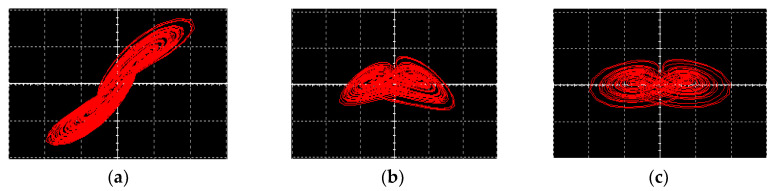

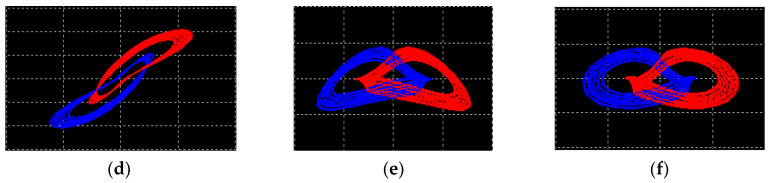
Simulation results obtained from the Multisim software. (**a**) Attractor on the *x*-*y* plane, (**b**) attractor on the *y*-*z* plane, (**c**) attractor on the *x*-*z* plane, (**d**) coexisting attractors the on *x*-*y* plane, (**e**) coexisting attractors on the *y*-*z* plane, (**f**) coexisting attractors on the *x*-*z* plane.

The simulation results obtained from oscilloscopes by the Multisim software are shown in Figure 15. Figure 15a–c are phase portraits of the attractors. Figure 15d–f are phase portraits of coexisting attractors, where the red orbit starts from the initial conditions of (0, 0.1, 0), and the blue orbit starts from (0, −0.1, 0). It is obvious that the simulation results matched well with the numerical simulation results.

## 6. Implementation of the Chaotic System by DSP Technology

The chaotic system can be used for digital encryption. The main characteristic of the proposed chaotic system is multistability. The multistability of the proposed system can enlarge the key space in encryption, which improves the encryption effect. Therefore, the proposed chaotic system can be used for digital encryption. In analog chaotic circuits, the existence of random signal perturbation will lead to output instability. So, the analog chaotic circuits cannot be directly applied to digital encryption. Therefore, in order to make the proposed chaotic system better suitable for digital encryption applications, it was further discretized and implemented by DSP technology [37].

During digital implementation, the dynamical degradation effect will eliminate the chaotic behavior in a finite space and prevent practical applications for chaos phenomena [43,44]. There are five approaches to prevent dynamical degradation: (1) higher finite precision [45]; (2) cascading multiple digital chaotic systems [46]; (3) perturbance-based method [47,48,49,50]; (4) switching multiple digital chaotic systems [51,52]; (5) error compensation methods [53]. In this paper, the first method was adopted to prevent dynamical degradation. The integration step in the discretization process is crucial.

There are three algorithms for discretization and digitalization of continuous chaotic systems. They are the simple Euler algorithm, the improved Euler algorithm, and the Runge–Kutta algorithm. The simple Euler algorithm has faster computation speed than the other two algorithms. When the Euler algorithm is implemented with DSP, it requires less resources and is easy to implement. Thus, the Euler algorithm was adopted to discretize the chaotic system. The theoretical basis of the Euler algorithm is shown as follows:

(18) $$f^{\prime }(x)=\underset{\Delta t\to 0}{\lim}\frac{x(t_{n}+\Delta t)-x(t_{n})}{\Delta t}=\underset{\Delta t\to 0}{\lim}\frac{x_{n+1}-x_{n}}{\Delta t}\approx \frac{x_{n+1}-x_{n}}{\Delta t}=\frac{x(n+1)-x(n)}{\Delta t}$$

Equation (8) can be discretized to the following equations according to the Euler algorithm:

(19) $$\left\{ \begin{array}{l} x(n+1)=(a(x(n)-y(n))+(m-n\left|z(n)\right|)x(n))\Delta t+x(n)\\ y(n+1)=(b(x(n)-y(n)))\Delta t+y(n)\\ z(n+1)=(-cz(n)-dx^{2}(n))\Delta t+z(n) \end{array}\right.$$

where the parameters *a*, *b*, *c*, *d*, *m*, and *n* are as in Table 1. The integration step **Δ***t* = 0.01 and the initial conditions were set as *x*(0) = 0, *y*(0) = 0.1, *z*(0) = 0. 

**Figure 16:**
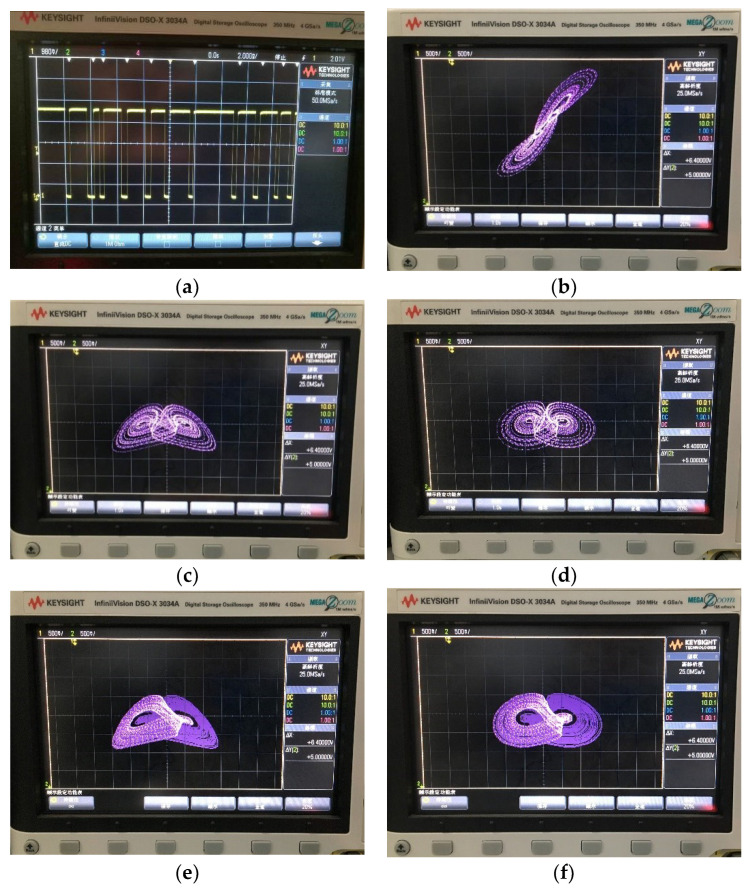
Experimental results obtained by Digital Signal Processing (DSP) technology. (**a**) Chaotic pseudo-noise (PN) sequence extracted from **the** variable *x*, (**b**) chaotic attractor on the *x*-*y* plane, (**c**) chaotic attractor on the *y*-*z* plane, (**d**) chaotic attractors on the *x*-*z* plane, (**e**) coexisting attractors on the *y*-*z* plane, (**f**) coexisting attractors on the *x*-*z* plane.

**Figure 17:**
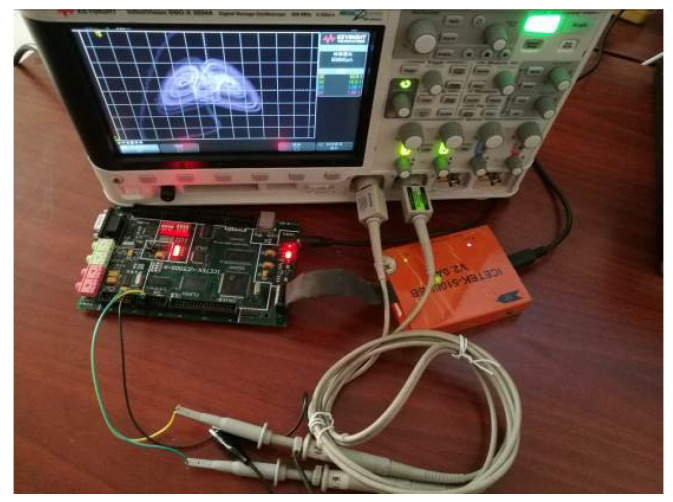
Experimental equipment.

The digital signal was obtained by solving Equation (19) in a digital signal processor. The chaotic pseudo-noise (PN) sequence extracted from the variable *x* is shown in Figure 16a. In order to observe the analog signal in an oscilloscope, the digital signal was input into an 8-bit D/A convertor. The output phase portraits of the chaotic attractor are shown in Figure 16b–d. The phase portraits of coexisting attractors are shown in Figure 16e,f, where the trajectory on the left starts from the initial conditions of (0, −0.1, 0), and the right trajectory starts from the initial conditions of (0, 0.1, 0). The experimental equipment is shown in Figure 17. In the experimental equipment, the type of evaluation board was ICETEK-VC5509-AE, and the core processing chip was TMS320C5509. 

## 7. NIST Test Results and Approximate Entropy Analysis of the Proposed Chaotic System

### 7.1. NIST Test Results 

The chaotic system can be used as a pseudo-random sequence generator to provide key sequences for an encryption system. The random characteristics of the chaotic sequences directly affect the security of an encryption system. The randomness of the binary sequences extracted from the above chaotic system were tested by means of the NIST test suite [38]. The NIST test suite is a tool for testing the randomness of pseudo-random sequences. 

In this NIST test, a binary sequence was generated from the solution sequence *z* of Equation (19). A binary sequence can be generated as follows: If the sixth place after the decimal point of a solution *z* is an even number, the generated binary number is 0, otherwise the generated binary number is 1. So, the solution sequence *z* can generate a binary sequence. In this NIST, the significance level *α* was set to 0.01. The binary sequence was divided into 1000 groups according to the significance level *α* [38], and each group contained 1,000,000 bits. The final test results are shown in Table 2.

In Table 2, two parameters evaluated in 15 tests are reported. One is the *p*-value_T_, which reflects the distribution of *p*-values (possible values). It is used to check for uniformity of the sequences [38]. If *p*-value_T_ ≥ 0.0001, the distribution of sequences is uniform [38]. It is obvious that the *p*-value_T_ of all types of test satisfied the above condition. The other parameter is the proportion, which represents the proportion of sequences that pass a statistical test [38]. The range of acceptable proportions is determined by the confidence interval. The range of acceptable proportions is computed as follows:

(20) $$\left(\hat{p}-3\sqrt{\hat{p}(1-\hat{p})/N},\hat{p}+3\sqrt{\hat{p}(1-\hat{p})/N}\right)$$

where $\hat{p}=1-\alpha$, *α* is the significance level, *N* is the total number of groups. In this test, *α* = 0.01, *N* = 1000, and the range of acceptable proportions was (0.9805608, 0.9994392). If the value of proportion is in the range of 0.9805608–0.9994392, the sequences pass this type of test [38]. It is obvious that all proportions were in the range of 0.9805608–0.9994392, which means that the sequences passed all types of test and the randomness of the proposed chaotic system was up to the standards of NIST [38]. Thus, when the integration step Δ*t* was set as 0.01 in Equation (19), the chaotic behavior was not eliminated. Therefore, the proposed chaotic system can be used as a pseudo-random sequence generator to provide key sequences for encryption systems.

### 7.2. Approximate Entropy Analysis

The purpose of the Approximate Entropy test is to measure the complexity and randomness of sequences [38]. In this NIST test, the chaotic sequences were divided into 1000 groups, and each group contained 1,000,000 bits. The Approximate Entropy test method is used to test the randomness of sequences in the following way:

(1) Construct a new sequence on the basis of each group of the original sequence. The method to construct the new sequence consists of appending *m*−1 bits (*m* is the block length) from the beginning of each group of the original sequence to the end of the original sequence.

(2) Count the frequency of all 2^m^ *m*-bit sub-sequences in the new sequence. Record the frequency of each *m*-bit sub-sequence as #*i* (*i* represents different *m*-bit sub-sequences).

(3) Compute $C_{i}^{m}$ as follows:

(21) $$C_{i}^{m}=\frac{\#i}{n}$$

where *m* represents the block length, *i* represents different *m*-bit strings, and *n* represents the length of each group of the new sequence.

(4) Compute *φ*^(*m*)^ as follows:

(22) $$\varphi^{\left(m\right)}=\sum_{j=0}^{2^{m}-1}C_{i}^{m}\ln C_{i}^{m}$$

(5) Compute *φ*^(*m*+1)^. Repeat steps (1)–(4) by replacing *m* with *m* + 1.

(6) Compute the test statistic χ^2^

(23) $$\chi^{2}=2n[\ln2-ApEn(m)]$$

where *ApEn*(*m*) = *φ*^(m)^ − *φ*^(m+1)^.

(7) Compute the *p*-value as follows:

(24) $$p\text{-}\mathrm{value}=igmc(2^{m-1},\frac{\chi^{2}}{2})$$

where *igamc* is the incomplete gamma function. It can be calculated as follows:

(25) $$igmc(a,x)=\frac{1}{\Gamma (a)}\int_{x}^{\infty }e^{-t}t^{a-1}dt$$

(26) $$\Gamma (a)=\int_{0}^{\infty }t^{a-1}e^{-t}dt$$

Since the chaotic sequences were divided into 1000 groups in this test, the number of *p*-values to compute was 1000.

(8) Compute *p*-value_T_ as follows:

(27) χ2=∑j=110(Fj−N10)2N10

(28) p-valueT=igmac(92,χ22)

where the intervals between 0 and 1 are divided into 10 sub-intervals; *j* represents one of the 10 sub-intervals, *F_i_* is the number of *p*-values in the sub-interval *j*, *N* is the total number of groups. In this test, *N* = 10,000. The Approximate Entropy test result is shown in Table 2. It is obvious that the Approximate Entropy test results were up to the standards of the NIST [38].

## 8. Conclusions

In this paper, an absolute voltage-controlled memristor model is proposed. The DC *V*–*I* plot analysis indicated that the proposed memristor was locally active. A simple Wien-bridge chaotic circuit based on the absolute memristor was designed. Because of the absence of an inductor, the above chaotic circuit was easily integrated. The presented chaotic circuit possesses rich dynamical behaviors, such as multistability and sustained chaos state. The simulation results of the chaotic circuit obtained by the Multisim software matched well with the numerical simulation results obtained by the Matlab software. The results of the DSP experiment and the NIST test indicated that the proposed chaotic system can be used as a pseudo-random sequence generator to provide key sequences for encryption systems. Therefore, the proposed chaotic system can be efficiently applied for digital information encryption. 

## Figures and Tables

**Table 1:** Parameter values of typical attractors.

Parameters	Values
*a*	4.5
*b*	5.5
*c*	0.4
*d*	1.0
*m*	5.0
*n*	4.0

**Table 2:** Final analysis report.

Statistical Test Terms	*p*-Value_T_	Proportion
Frequency	0.624627	0.9960
Block Frequency	0.668321	0.9940
Cumulative Sums	0.326749	0.9960
Runs	0.399442	0.9900
Longest Run	0.877083	0.9880
Rank	0.044797	0.9900
FFT	0.887645	0.9860
Non-Overlapping Template	0.993493	0.9880
Overlapping Template	0.476911	0.9930
Universal	0.854708	0.9870
Approximate Entropy	0.272977	0.9890
Random Excursions	0.649066	0.9935
Random Excursions Variant	0.995975	0.9951
Serial	0.007805	0.9820
Linear Complexity	0.755819	0.9920
